# Age-Associated ALU Element Instability in White Blood Cells Is Linked to Lower Survival in Elderly Adults: A Preliminary Cohort Study

**DOI:** 10.1371/journal.pone.0169628

**Published:** 2017-01-06

**Authors:** R. Garrett Morgan, Massimo Venturelli, Cole Gross, Cantor Tarperi, Federico Schena, Carlo Reggiani, Fabio Naro, Anna Pedrinolla, Lucia Monaco, Russell S. Richardson, Anthony J. Donato

**Affiliations:** 1 Department of Internal Medicine, Division of Geriatrics, University of Utah School of Medicine, University of Utah, Salt Lake City, Utah, United States of America; 2 Geriatric Research, Education, and Clinical Center, George E. Wahlen Department of Veterans Affairs Medical Center, Salt Lake City, Utah, United States of America; 3 Department of Neurological and Movement Sciences, University of Verona, Verona, Italy; 4 Department of Biomedical Sciences for Health, University of Milan, Milan, Italy; 5 Department of Biomedical Sciences, University of Padova, Padova, Italy; 6 DAHFMO-Unit of Histology and Medical Embryology, Sapienza University, Rome, Italy; 7 Department of Medicine, University of Verona, Verona, Italy; 8 Department of Physiology and Pharmacology, Sapienza University of Rome, Italy; 9 Department of Health, Kinesiology, and Recreation, University of Utah, Salt Lake City, Utah, United States of America; Universitat des Saarlandes, GERMANY

## Abstract

**Background:**

ALU element instability could contribute to gene function variance in aging, and may partly explain variation in human lifespan.

**Objective:**

To assess the role of ALU element instability in human aging and the potential efficacy of ALU element content as a marker of biological aging and survival.

**Design:**

Preliminary cohort study.

**Methods:**

We measured two high frequency ALU element subfamilies, *ALU-J* and *ALU-Sx*, by a single qPCR assay and compared *ALU-J/Sx* content in white blood cell (WBCs) and skeletal muscle cell (SMCs) biopsies from twenty-three elderly adults with sixteen healthy sex-balanced young adults; all-cause survival rates of elderly adults predicted by *ALU-J/Sx* content in both tissues; and cardiovascular disease (CVD)- and cancer-specific survival rates of elderly adults predicted by *ALU-J/Sx* content in both tissues, as planned subgroup analyses.

**Results:**

We found greater *ALU-J/Sx* content variance in WBCs from elderly adults than young adults (*P* < 0.001) with no difference in SMCs (*P* = 0.94). Elderly adults with low WBC *ALU-J/Sx* content had worse four-year all-cause and CVD-associated survival than those with high *ALU-J/Sx* content (both *P* = 0.03 and hazard ratios (HR) ≥ 3.40), while WBC *ALU-J/Sx* content had no influence on cancer-associated survival (*P* = 0.42 and HR = 0.74). SMC *ALU-J/Sx* content had no influence on all-cause, CVD- or cancer -associated survival (all *P* ≥ 0.26; HR ≤ 2.07).

**Conclusions:**

These initial findings demonstrate that ALU element instability occurs with advanced age in WBCs, but not SMCs, and imparts greater risk of all-cause mortality that is likely driven by an increased risk for CVD and not cancer.

## Introduction

Aging is associated with variance in gene function between individuals, which can result from somatic mutation, and may partly underlie variation in human lifespan[1, 2]. While their role in age-associated somatic mutation and mortality is unknown, a key characteristic of many mutations linked to inherited disease in humans is the involvement of retrotransposons, such as ALU elements, which seem to facilitate mutational events[3]. Indeed, ALU element instability is thought to be responsible for 0.1% of all genetic diseases in humans[3]. ALU elements, named after the restriction enzyme (*AluI*), are the most abundant retrotransposons in the human genome and comprise 10–15% of genome mass[4]. They consist of several subfamilies, the oldest of which, *ALU-Sx* and *ALU-J*, represent nearly 80% of all ALU elements[4, 5]. ALU elements are distributed randomly across all chromosomes; however, they tend to cluster near genes in introns, UTRs, and various intergenic regions, which means that ALU element instability frequently results in alterations in gene function[3, 4].

ALU-mediated sequence deletions and insertions can occur through the process of non-allelic homologous recombination (NAHR) during repair of DNA damage[3, 5], or fork stalling and template switching (FoSTeS)/microhomology-mediated break-induced replication (MMBIR) during DNA synthesis[6–8]. ALU-mediated genomic rearrangements in germ cells have been linked to several chronic diseases, including, Parkinson’s disease, breast and ovarian cancer, and pulmonary artery hypertension[9–13]. Genome wide copy number and inter-ALU proximity are the greatest contributing factors for ALU element mediated NAHR[5]. Consequently, *ALU-Sx* and *ALU-J* are involved in over 70% of all known ALU-mediated NAHR deletion events in the human genome[5]. ALU-mediated sequence insertions can also occur through retrotransposition, whereby ALU elements are reverse transcribed and randomly inserted back into the genome [3, 4] Retrotransposition rates are thought to increase over time following age-related hypomethylation of ALU elements[14–16]; however, the retrotransposition rates of older subfamilies, like *ALU-Sx* and *ALU-J*, are predicted to be far lower than that of younger subfamilies[17–19]. Interestingly, age-related hypomethylation of ALU elements has been linked to osteoporosis, obesity, and gastric and lung cancer[15, 16, 20–22]. Importantly, clonal selection and expansion of mutant genomes in proliferative cell types might amplify any maladaptive effects within a tissue following an ALU-mediated mutational event[1, 23, 24].

ALU element instability, or variance in ALU element copy number, may occur over time through the processes of NAHR, FoSTeS/MMBIR and clonal expansion; contribute to variance in gene function with advancing age; and partly explain variation in human lifespan. The role of ALU element instability in human aging and lifespan has been previously suggested[25], but to date has not been directly investigated. To conduct the first assessment of the role of ALU element instability in human aging and the potential efficacy of ALU element content as a marker of biological aging and survival, we measured ALU element content using a high throughput technique in white blood cells (WBCs) and skeletal muscle cells (SMCs) from young and elderly adults. We then compared ALU element content between young and elderly adults in each tissue; all-cause survival rates of elderly adults predicted by *ALU-J/Sx* content in both tissues; and cause-specific survival rates of elderly adults predicted by *ALU-J/Sx* content in both tissues. Cause-specific survival analyses were conducted for the two leading causes of death among our participants, cardiovascular disease (CVD) and cancer, as planned subgroup analyses. Here we demonstrate that ALU element instability occurs with advanced age in WBCs, but not SMCs, and imparts greater risk of all-cause mortality that is likely driven by an increased risk for CVD rather than cancer.

## Materials and Methods

### Study design and participants

We conducted a preliminary cohort study in elderly adults with age-group comparisons between young and elderly participants. Two separate tissue samples (WBCs and SMCs) were obtained from twenty-three elderly adults and sixteen healthy young adults without a history of smoking. All elderly participants were 75 yrs or older and young participants were 30 yrs or younger to minimize within group influences of age on outcomes. All participants were unrelated, but ethnically similar, white Europeans of Italian descent and elderly and young adult age-groups were frequency-balanced for sex to account for the potential influence of ethnicity and sex differences on outcomes. The elderly participants were community dwelling residents of Mantua, Italy, and patients at the Mons Mazzali Geriatric Institute of Mantua; young adult participants were comprised of college students at the University of Verona; and all participants were recruited through public advertisement with flyers between December 2010 and May 2011[26]. Elderly adult mortality and morbidity was tracked for four years by annual medical record audits from clinics at the Mons Mazzali Geriatric Institute of Mantua. Primary outcomes included *ALU-J/Sx* content standard deviation (variance) and mean differences between age-groups and tissues, and all-cause survival rates of elderly adults predicted by *ALU-J/Sx* content. Secondary outcomes included variance and mean differences in *ALU-J/Sx* PCR product melting temperature, *ALU-J/Sx* PCR product melting peak width, mean telomere length, and four-year cause-specific survival rates of elderly adults predicted by *ALU-J/Sx* content in each tissue. Cause-specific survival comparisons were conducted with the two leading causes of death among our participants, CVD and cancer, as planned subgroup analyses. Only participants with *ALU-J/Sx* content data from both WBCs and SMCs were used for tissue comparisons (elderly adult: n = 19; young adult: n = 10), while all available data was used for age-group comparisons. Likewise, only elderly adults with *ALU-J/Sx* content data from both WBCs and SMCs were used for survival analyses. All surviving subjects were included in each analysis as censured data. Low vs high *ALU-J/Sx* content group N’s and *ALU-J/Sx* content ranges for each survival analysis are included below in Table 1. This study was conducted in accordance with the Declaration of Helsinki (2008) of the World Medical Association; the Institutional Review Boards of the University of Verona, University of Utah, and the Salt Lake City Veteran’s Affairs Medical Center approved all protocols; and written informed consent was obtained from all participants and/or family caregivers prior to tissue sample collection.

**Table 1:** Survival analyses *ALU-J/Sx* content group N’s and *ALU-J/Sx* content ranges.

	WBC		SMC	
	Low	High	Low	High
**All-cause survival**				
N	9	10	9	10
*ALU-J/Sx* range (a.u.)	0.70–1.0	1.11–3.41	0.58–0.99	1.14–1.52
**CVD-specific survival**				
N	8	8	8	8
*ALU-J/Sx* range (a.u.)	0.72–1.0	1.11–3.41	0.60–0.99	1.14–1.52
**Cancer-specific survival**				
N	5	6	5	6
*ALU-J/Sx* range (a.u.)	0.73–1.11	1.15–3.41	0.58–0.98	0.99–1.46

Terms—WBC: white blood cells; SMC: skeletal muscle cells; *ALU-J/Sx*: *ALU-J* and *ALU-Sx* content; CVD: cardiovascular disease.

### Tissue collection

Anonymized tissue samples were collected in clinics at the Mons Mazzali Geriatric Institute of Mantua and University of Verona between December 2010 and May 2011. Some participants were unable to donate SMC samples, thus more WBC samples (elderly adult: n = 22; young adult: n = 14) were obtained than SMC samples (elderly adult: n = 20; young adult: n = 12) within each age-group. A WBC-enriched fraction of whole blood was prepared by centrifuging whole blood at 2500 x g for 10 minutes at room temperature. After centrifugation, the intermediate buffy coat layer containing concentrated WBCs was collected. Skeletal muscle biopsies were obtained with a 14-gauge tru-cut needle from the *vastus lateralis*. All tissue samples were stored at -80°C until processing and analysis in the Translational Vascular Physiology Laboratory at the University of Utah and Veteran’s Affairs Medical Center-Salt Lake City, Geriatric Research Education and Clinical Center, between July 2012 and November 2013.

### *ALU-J/Sx* content analysis

To assess general ALU element instability with a single assay, we chose to measure *ALU-J* and *ALU-Sx* by qPCR, as these ALU elements represent older subfamilies with a relatively high genome wide copy number and sufficient sequence similarity to be targeted with a single PCR primer pair. Total genomic DNA was isolated and purified from all tissue samples using QIAamp mini DNA kit (Qiagen, Inc.) according to the manufacturer’s protocol. Sequence-independent qPCR with RT^2^ SYBR Green qPCR Mastermix (Qiagen, Inc.) was performed according to the manufacturer’s protocol to determine relatively quantitative standard curve derived *ALU-J/Sx* and *albumin* content in all samples. Relatively quantitative standard curves were generated by dilution series of standard pool human DNA. *Albumin* was used as single copy gene to control for genome content in samples, and *ALU-J/Sx* content per genome was expressed as the ratio of *ALU-J/Sx* to *albumin* content.

To simultaneously capture both *ALU-J* and *ALU-Sx* elements, primers were designed to target regions that were as close to identical in sequence as possible in the respective ALU element consensus sequences, and would produce PCR products of roughly the same length (*ALU-J*: 93 bp vs. *ALU-Sx*: 83 bp). The forward primer targeted identical sequences in the same regions of both *ALU-J* and *ALU-Sx* consensus sequences, and the reverse primer matched the sequence in its corresponding region of the *ALU-J* consensus sequence, but contained two mismatches with the sequence in the same region of the *ALU-Sx* consensus sequence at positions 9 and 16 as highlighted below. Cycling conditions were adjusted to allow stability of the *ALU-Sx* reverse primer despite the mismatches, as determined by PrimerQuest® Tool. To capture the regions of *ALU-J* and *ALU-Sx* elements most likely to be involved in NAHR, primers were designed to flank the region of the respective consensus sequences that is predicted to be most prone to breakage (5’ to poly(A) core; as highlighted below)[5]. *Albumin* was chosen as the single copy gene because it is a relatively ALU-poor gene, as determined by BLAST, which should minimize the effects of ALU element instability on single copy gene signal. As a further precaution, primers were designed to generate a product from a non-ALU containing region of the *albumin* gene. Primer sequences were as follows; *ALU-J/Sx*: fwd-TCACGCCTGTAATCCCAGCACTTT, rev-GGTTTCACTATGTTGCCCAGGCT and *albumin*: fwd-AAATGCTGCACAGAATCCTTG, rev-GAAAAGCATGGTCGCCTGTT. *ALU-J* and *ALU-Sx* consensus sequences with primer binding sites highlighted in bold and mismatches within *ALU-Sx* consensus sequence represented as underlined lower case letters include:

#### *ALU-J* (gi: 551536)

ggccgggcgcggtggc***TCACGCCTGTAATCCCAGCACTTT***gggaggccgaggcgggaggatcacttgagcccaggagttcgagacc***AGCCTGGGCAACATAGTGAAACC***ccgtctctacaaaaaatacaaaaattagccgggcgtggtggcgcgcgcctgtagtcccagctactcgggaggctgaggcaggaggatcgcttgagcccgggaggtcgaggctgcagtgagccgtgatcgcgcactgcactccagcctgggcgacagagcgagaccctgtctcaaaaaaaa.

#### *ALU-Sx* (gi: 551543)

ggcgggcggaggccgggcgcggtggc***TCACGCCTGTAATCCCAGCACTTT***gggaggaagatcacctgaggtcaggagttcgagacc***AGCCTGG******c******CAACAT******g******GTGAAACC***ccgtctctactaaaaatacaaaaattagccgggcgtggtggcgcgcgcctgtaatcccagctactcgggaggctgaggcaggagaatcgcttgaacccgggaggcggaggttgcagtgagccgagatcgcgccactgcactccagcctgggcgacagagcgagactccgtctcaaaaaaaa.

All samples were assayed in triplicate, and replicate means were used for analysis. Two samples (young adult WBC: n = 1; elderly adult SMC: n = 1) did not produce valid qPCR signals and were discarded. All utilized replicates were ≤ 0.5 threshold cycles apart, and standard curves had correlation coefficients (R^2^) of ≥ 0.98. The intra-assay coefficient of variation for all *ALU-J/Sx* measures was 13.1%, and inter-assay coefficient of variation, calculated using standard curves from each plate, was 1.2%. Both values are within the range considered acceptable for quantitative PCR experiments[27].

### *ALU-J/Sx* size analysis

Melting curve analysis was performed on all samples following completion of the PCR program to assess *ALU-J/Sx* sizes[28]. When gradually heated, the temperatures at which PCR products melt, or lose ~50% of SYBR green signal, expressed as relative fluorescence units (RFUs), directly corresponds to the size of the PCR product. The larger the melting temperature, the larger the PCR product. The negative rate of change in RFUs as the temperature (T) changes during melting program (–d/(RFU)/dT) can be plotted to generate peaks that directly correspond to melted PCR products. The width of melting peaks can be analyzed to determine the homogeneity of PCR product sizes within a given sample. The wider the peak and/or range of peaks, the more heterogeneous the sizes of PCR products are within a sample.

### Mean telomere length (chromosomal content) analysis

To estimate chromosomal content, we measured mean telomere length (mTL) as we have previously described at length[28–30]. We believe mTL represents a good estimate of chromosome content, as telomeres are present on every chromosome. Furthermore, telomeres contain the same base sequence on each chromosome to facilitate analysis by qPCR without requiring assessment of 23 unique chromosomal markers. In brief, a sequence-independent multiplex qPCR technique using a SYBR Green master mix with 0.625U AmpliTaq Gold 360 DNA polymerase (Life Technologies Corporation) was utilized to determine mTL as described by Cawthon[28]. Telomeric DNA (T) content and *albumin* content, used as single copy gene (S) to control for cell concentration in samples, were generated by relatively quantitative standard curve and mean telomere content per cell was expressed as the T/S ratio. mTLs per cell were generated by converting T/S ratios to bp of DNA using the formula: bp *=* 3330(T/S) + 3730, derived by Cawthon[28]. The intra- and inter-assay coefficient of variation for all mTL measures and plates was 10.5% and 0.6%, respectively, which is within the range considered acceptable for quantitative PCR experiments[27, 28].

### Data analysis

F-tests and unpaired two-tailed t-tests with unequal variances assumed were used to assess variance and mean differences, respectively, in all outcomes. Unpaired two-tailed t-tests with equal variances assumed were used to compare means of continuous variable participant characteristics between age-groups, and two-tailed Chi-squared tests were used to compare categorical participant characteristics where appropriate. Kaplan-Meier survival analyses were conducted to create and compare all-cause and cause-specific (CVD and cancer) survival rates between elderly adults with low *ALU-J/Sx* content (lower 50^th^ percentile of elderly adult *ALU-J/Sx* content) and high *ALU-J/Sx* content (upper 50^th^ percentile of elderly adult *ALU-J/Sx* content) in WBCs and SMCs. The product limit method was used to create survival curves and one-tailed Mantel-Cox log-rank tests were used to compare curves. Hazard ratios (HRs) were calculated by log-rank method from the slopes of survival curves and used to compare the rate at which participants with low *ALU-J/Sx* content died to that which those with low *ALU-J/Sx* content died. 95% confidence intervals (CIs) were calculated for all HRs to estimate effect accuracy in survival analyses. Only elderly adults with *ALU-J/Sx* content data from both WBCs and SMCs were used for survival analyses. All surviving subjects were included in each analysis as censure data. All data were tested for normality by the D’Agostino-Pearson omnibus normality test. Significance was set at *P <* 0.05 and all analyses were completed using GraphPad Prism version 6.0.

#### Sample size justification

The primary sample size determinant was the willingness and ability of study participants to donate skeletal muscle biopsies. This limited our sample size, but as outlined below, did not adversely impact the accuracy of our results. As no other studies have measured age-associated ALU element content changes, we could not perform a reliable *a priori* power analysis to determine the number of participants needed to observe an accurate effect. Thus, we conducted *post hoc* analyses to determine whether the observed effects in our samples accurately represented that in the population our samples were drawn from. The most reliable *post hoc* test for this purpose is an assessment of CI overlap for the variables of interest[31–34]. If a difference was observed for a variable of interest compared between two samples and the CIs (set at confidence level that corresponds to difference of desired alpha; i.e. assuming unequal variance: ~86% CI corresponds to α = 0.05) for that variable in both samples do not overlap, then the observed sample difference accurately reflects the true difference in the relevant population[31–34]. Thus, the number of participants in samples utilized for the statistical test was sufficient to detect the observed difference and a Type I error did not occur[31–34]. The inverse is true for non-significant results[31–34]. Thus, we assessed the overlap of CIs of our primary variable of interest, standard deviations (SDs), of *ALU-J/Sx* content between age-groups. To be conservative, we calculated the overlap of 99% CIs to determine likelihood that we committed a Type I error and overlap of 90% CIs to determine likelihood that we committed a Type 2 error due to inadequate sample sizes.

## Results

We measured total *ALU-J*/*Sx* content in WBCs and SMCs from twenty-three elderly adults (mean ± SD: 85 ± 4 yrs) and sixteen healthy young adults (mean±SD: 25±2 yrs) without a history of smoking. All-cause and cause-specific survival of elderly adults was tracked for four years, with complete follow-up data collected for all participants. Narrow age-ranges were maintained within each age-group to minimize within group influences of age on outcomes. All participants were unrelated, but ethnically similar, white Europeans of Italian descent, and elderly and young adult groups were sex-balanced (age-group sex distribution difference: *P* = 0.31) to account for the potential influence of ethnicity and sex on outcomes (participant characteristics presented in Table 2). Furthermore, all participants were self-selected; however, the observed participant characteristics were similar to what might be expected in these respective age-groups in the general population. Thus, we found no evidence that self-selection influenced our findings.

**Table 2:** Participant characteristics.

Characteristic	Young Adults (n = 16)	Elderly Adults (n = 23)
Age (yrs)^a^	25 ± 2	85 ± 4
Male^b^	6 (37%)	5 (22%)
Female^b^	10 (63%)	18 (78%)
BMI (kg/m^2^)^a^	23 ± 2	22 ± 2
SBP (mmHg)^a^	120 ± 5	130 ± 8
DBP (mmHg)^a^	80 ± 3	86 ± 2
Glucose (mg/dL)^a^	86 ± 6	96 ± 4
Hb (g/dL)^a^	13.2 ± 0.6	11.4 ± 0.3
HDL (mg/dL)^a^	51 ± 2	53 ± 3
LDL(mg/dL)^a^	96 ± 4	110 ± 5
TDEE (kcal/day)^a^	1927 ± 200	1483 ± 242
***Medical history***		
Cancer^b^	0 (0%)	6 (26%)
CVD^b^	0 (0%)	13 (57%)
DM2^b^	0 (0%)	4 (17%)
COPD^b^	0 (0%)	2 (9%)

Data presented are ^a^mean ± SD and ^b^n (%) within age-groups. Terms—BMI: body mass index, SBP: systolic blood pressure, DBP: diastolic blood pressure, TDEE: total daily energy expenditure, CVD: cardiovascular disease, DM2: type 2diabetes mellitus, and COPD: chronic obstructive pulmonary disease.

**Fig 1:**
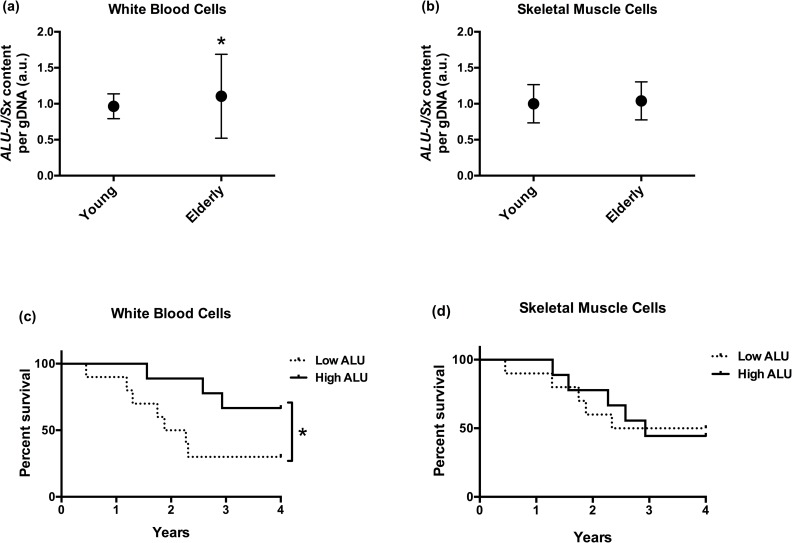
Age-associated ALU element instability and survival. **(a, b)** *ALU-J/Sx* content per genome normalized to young adult group mean with SDs presented (F-test: ^★^*P* < 0.001), and **(c, d)** percent survival of elderly adults predicted by *ALU-J/Sx* content over four years (^★^*P* = 0.03; WBC-HR = 3.40, CI = 0.95–12.18; SMC-HR = 1.02, CI = 0.30–3.54). Terms—gDNA: genomic DNA; SD: standard deviation; *ALU-J/Sx* content: combined *ALU-J* and *ALU-Sx* content; WBC: white blood cells; SMC: smooth muscle cells; HR: hazard ratio; CI: confidence interval.

*ALU-J/Sx* content variance and means were compared between young and elderly adults in each tissue. We found greater *ALU-J/Sx* content variance, but no mean difference, in WBCs from elderly adults than those from young adults (*P* < 0.001 and 0.31, respectively; Fig 1A), and no age-group variance or mean differences in SMCs (both *P* ≥ 0.68; Fig 1B). We found greater *ALU-J/Sx* content variance, and no mean difference, in WBCs compared with SMCs from elderly adults (*P* < 0.001 and 0.33, respectively), but no inter-tissue variance or mean differences in young adults (both *P* > 0.05). Evaluation of effect accuracy, and thus sample size adequacy, was confirmed by assessing overlap of 90% and 99% CIs of all age-group SDs (presented in Table 3). Melting curve analysis revealed no differences in variance in average *ALU-J/Sx* PCR product size or distribution of sizes between age-groups (all *P* ≥ 0.13). We found no evidence of age-associated abnormalities in chromosome content, as there were no differences in mTL variance between age-groups (both *P* ≥ 0.54), and all measured mTLs were very consistent with those reported in studies of normal age-associated telomere lengths (elderly adult mTL ± SD: WBC-5955 ± 1081 bp and SMC-12,545 ± 2029 bp)[35–37]. Thus, age-associated ALU element instability in WBCs is likely due primarily to variance in copy number and not differences in mean ALU element size or age-associated chromosomal instability in our elderly participants, which supports the overall hypothesis that ALU element instability *per se* occurs with advanced age.

**Table 3:** Assessment of 90% and 99% CI overlap of age-group comparison SDs.

	90% CI		99% CI	
	WBCs	SMCs	WBCs	SMCs
**Young Adults**	0.14–0.27	0.20–0.42	0.12–0.34	0.17–0.56
**Elderly Adults**	0.49–0.82^★^	0.21–0.36^**†**^	0.43–0.99^★^	0.19–0.44^**†**^

Data presented are 90% and 99% CIs of SDs for *ALU-J/Sx* content (^★^no overlap of 90% or 99% CI between young and elderly adults within respective tissue; reported *P* < 0.001; thus no Type I error/^**†**^overlap of 90% CI between young and elderly adults within respective tissue; reported *P* > 0.05; thus no Type II error). Terms–SD: standard deviations; CI: confidence interval; WBC: white blood cells; SMC: skeletal muscle cells; *ALU-J/Sx*: *ALU-J* and *ALU-Sx* content.

**Fig 2:**
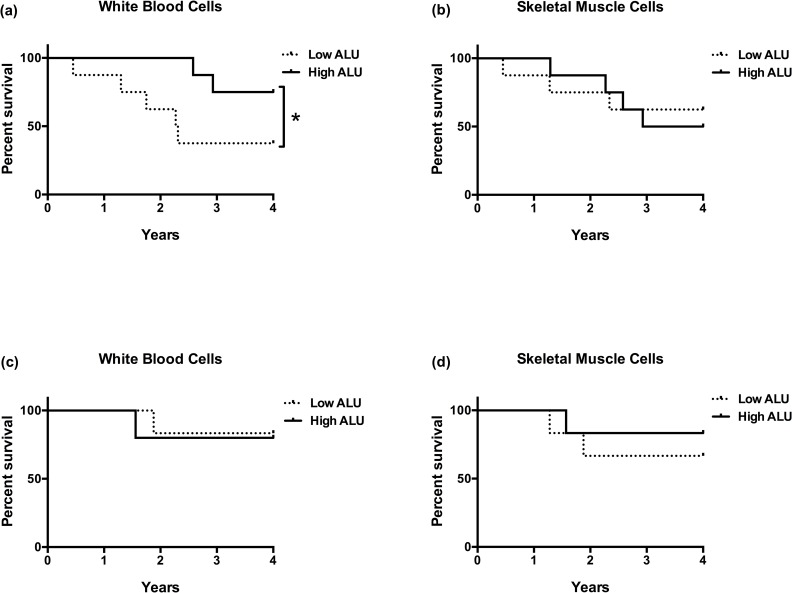
ALU element instability and cause-specific survival. **(a, b)** CVD-associated percent survival of elderly adults predicted by *ALU-J/Sx* content over four years (^★^*P* = 0.03; WBC-HR = 4.21, CI = 0.90–19.70; SMC-HR = 0.84, CI = 0.19–3.70), and **(c, d)** cancer-associated percent survival of elderly adults predicted by *ALU-J/Sx* content over four years (WBC-HR = 0.74, CI = 0.05–12.28; SMC-HR = 2.07, CI = 0.22–20.00). Terms—CVD: cardiovascular disease; *ALU-J/Sx* content: combined *ALU-J* and *ALU-Sx* content; WBC: white blood cells; SMC: smooth muscle cells; HR: hazard ratio; CI: confidence interval.

Four-year survival rates were compared between elderly adults in the lower (low) and upper (high) 50^th^ percentiles of *ALU-J/Sx* content in both tissues. We found that elderly adults with low WBC *ALU-J*/Sx content had worse all-cause survival than those with high *ALU-J*/Sx content (*P* = 0.03; HR = 3.40; HR CI = 0.95–12.18; Fig 1C); but there was no difference in all-cause survival between elderly adults with low and high SMC *ALU-J*/Sx content (*P* = 0.49; HR = 1.02; HR CI = 0.30–3.54; Fig 1D). Additionally, we compared four-year cause-specific survival rates between elderly adults with low and high *ALU-J*/Sx content that died from CVD and cancer in each tissue. Among elderly adults that succumbed to CVD, we found that those with low *ALU-J*/Sx content in WBCs had worse survival than those with high *ALU-J*/Sx content (*P* = 0.03; HR = 4.21; HR CI = 0.90–19.70; Fig 2A); while there was no difference in CVD-associated survival between elderly adults with low and high SMC *ALU-J*/Sx content (*P* = 0.41 and HR = 0.84; HR CI = 0.19–3.70; Fig 2B). Among those that succumbed to cancer, there were no differences in four-year survival rates between those with low and high *ALU-J*/Sx content in either WBCs or SMCs (both *P* ≥ 0.26; WBC-HR = 0.74, CI = 0.05–12.28; SMC-HR = 2.07, CI = 0.22–20.00; Fig 2C and 2D). There were no differences in age or sex distribution between low and high *ALU-J*/Sx content groups in any survival comparison (all *P* ≥ 0.14).

## Discussion

This preliminary study was designed to assess the role of ALU element instability in human aging and the potential efficacy of ALU element content as a marker of biological aging and survival, as well as establish the rationale for conducting a cohort study using similar techniques in larger more diverse samples observed over a longer period of time. Comparisons of ALU element instability in two separate tissues allowed us to assess whether variance in ALU element copy number occurred somatically. Furthermore, we chose tissues that are clinically relevant to aging and chronic disease, but experience different levels of DNA damage over time and have differing proliferative potentials, which should provide insight into possible mechanisms that influence ALU element instability and guide future experimental studies aimed at describing these mechanisms. SMCs are under relatively greater oxidative genotoxic stress than WBCs, which might lead to relatively more DNA damage and genomic instability[26]; however, WBCs are a highly proliferative cell population compared with SMCs and are thus under greater replicative stress and more capable of clonal selection and expansion of mutant genomes[1, 23, 24].

These initial findings demonstrate that ALU element instability occurs with advanced age in WBCs and not SMCs, which suggests that replication-dependent mutational processes, like FoSTeS/MMBIR, and the capacity for clonal expansion of mutant genomes may be more influential factors for inter-individual variance in ALU element copy number than DNA damage rates. Indeed, there is evidence that ALU elements mediate complex structural mutations through FoSTeS/MMBIR by providing nearby complementary targets for disassociated ALU-containing templates following replication fork stalling or collapse that are separated by sizeable linear distance[6–8]. In addition to deletion or insertion of the base sequence between ALUs, this process would also result in alterations in ALU element copy number. Likewise, a somatic selection event following an ALU-mediated mutation that results in clonal expansion of a mutant cell would increase the frequency of genomes with altered ALU element copy number within a tissue[1, 24]. This might partly explain the observed inter-individual variance in WBC *ALU-J/Sx* content in elderly adults, as different individuals may have clonally expanded mutant genomes over time with different alterations in ALU element content. Importantly, these divergent results in different tissues provide evidence that age-associated ALU element instability occurs somatically in WBCs. Furthermore, ALU-mediated deletions in WBCs, but not SMCs, impart greater risk of all-cause mortality that may be driven by an increased risk for CVD and not cancer. The role of WBCs, which are comprised of immune cells, in arterial inflammation and plaque formation has long been established[38]. Thus, ALU element instability mediated gene function alterations in WBCs may tend to lead to a pro-inflammatory phenotype and/or enhanced immune cell infiltration into the arterial wall, and subsequently promote CVD pathogenesis.

The observed age-group and survival differences are assumed to apply to the general population and beyond the observation time of four years; however, we believe these preliminary results must be verified by a larger scale study. An important consideration is that our smaller sample sizes preclude a comprehensive assessment of confounders and effect modifiers within our data, such as sex, cause of death, and birth cohort. We attempted to account for some of these effects by maintaining sex-balanced age-groups with relatively narrow age-ranges, and conducting planned subgroup analyses to assess cause-specific survival. However, a thorough assessment of the influence of sex, cause of death, and birth cohort on these findings must be explored in a study with larger more diverse samples observed over longer periods of time. Additionally, while we believe our sample sizes were sufficient to accurately detect the observed age-group difference in WBCs based on the assessment of CI overlap, a few high or low data points could drive group differences in variance with small samples. Importantly, removal of the highest and lowest one or two *ALU-J/Sx* content values from the WBC age-group comparison still yields an age-associated variance difference (*P* < 0.01).

An interesting finding from this study was that mean WBC *ALU-J/Sx* content was not different between young and elderly adults, despite the observation that elderly adults with lower WBC *ALU-J/Sx* content had worse all-cause survival than those with higher WBC *ALU-J/Sx* content. One might expect mean WBC *ALU-J/Sx* content to increase over time as individuals with lower *ALU-J/Sx* content are selected out. We indeed found that elderly adults had modestly elevated (14%) WBC *ALU-J/Sx* content compared with young adults. Additionally, among elderly adults that survived the four year study period, WBC *ALU-J/Sx* content was 44% higher than that in young adults; however, this difference did not reach significance (*P* = 0.08). In light of these findings, we can’t rule out the possibility that by studying elderly adults we captured the life stage that ALU element instability has the most influence on mortality. This could result from reaching a critical threshold of ALU element instability with advanced age, after which survival is impacted, or a burst of genomic instability in elderly adults due to breakdown of DNA repair/replication systems or anti-oxidant defenses. This possibility, as well as the exclusion of middle-aged adults from our study, limits the external validity of these observations to young and elderly adults. Assessment of ALU element instability in middle-aged adults and its corresponding impact on survival must be addressed in a study with samples of a broader age-range.

## Conclusions

We believe these findings provide sufficient rationale for conducting larger cohort studies using similar techniques to validate ALU element instability in WBCs as a marker of biological aging and determinant of human lifespan. Furthermore, this study provides the clinical foundation for conducting experimental studies aimed at describing mechanisms by which replicative stress and clonal expansion might lead to ALU element instability in proliferative cell types and how WBC ALU element instability might mediate all-cause and CVD-associated mortality.

## Supporting Information

S1 File Minimal Datasets from Findings. (XLSX)

## References

[1] Vijg J. Somatic mutations, genome mosaicism, cancer and aging. Curr Opin Genet Dev. 2014;26:141–9. 10.1016/j.gde.2014.04.002 25282114 PMC5480293

[2] Martin GM. Epigenetic gambling and epigenetic drift as an antagonistic pleiotropic mechanism of aging. Aging Cell. 2009;8(6):761–4. 10.1111/j.1474-9726.2009.00515.x 19732045

[3] Deininger PL, Batzer MA. Alu repeats and human disease. Mol Genet Metab. 1999;67(3):183–93. Epub 1999/06/25. 10.1006/mgme.1999.2864 10381326

[4] Batzer MA, Deininger PL. Alu repeats and human genomic diversity. Nat Rev Genet. 2002;3(5):370–9. Epub 2002/05/04. 10.1038/nrg798 11988762

[5] Sen SK, Han K, Wang J, Lee J, Wang H, Callinan PA, et al Human genomic deletions mediated by recombination between Alu elements. Am J Hum Genet. 2006;79(1):41–53. PubMed Central PMCID: PMCPMC1474114. 10.1086/504600 16773564 PMC1474114

[6] Bondurand N, Fouquet V, Baral V, Lecerf L, Loundon N, Goossens M, et al Alu-mediated deletion of SOX10 regulatory elements in Waardenburg syndrome type 4. Eur J Hum Genet. 2012;20(9):990–4. PubMed Central PMCID: PMCPMC3421117. 10.1038/ejhg.2012.29 22378281 PMC3421117

[7] Choi BO, Kim NK, Park SW, Hyun YS, Jeon HJ, Hwang JH, et al Inheritance of Charcot-Marie-Tooth disease 1A with rare nonrecurrent genomic rearrangement. Neurogenetics. 2011;12(1):51–8. 10.1007/s10048-010-0272-3 21193943

[8] Stankiewicz P, Sen P, Bhatt SS, Storer M, Xia Z, Bejjani BA, et al Genomic and genic deletions of the FOX gene cluster on 16q24.1 and inactivating mutations of FOXF1 cause alveolar capillary dysplasia and other malformations. Am J Hum Genet. 2009;84(6):780–91. PubMed Central PMCID: PMCPMC2694971. 10.1016/j.ajhg.2009.05.005 19500772 PMC2694971

[9] Yang C, Arnold AG, Trottier M, Sonoda Y, Abu-Rustum NR, Zivanovic O, et al Characterization of a novel germline PALB2 duplication in a hereditary breast and ovarian cancer family. Breast Cancer Res Treat. 2016;160(3):447–56. 10.1007/s10549-016-4021-7 27757719 PMC5646170

[10] Kataoka M, Aimi Y, Yanagisawa R, Ono M, Oka A, Fukuda K, et al Alu-mediated nonallelic homologous and nonhomologous recombination in the BMPR2 gene in heritable pulmonary arterial hypertension. Genet Med. 2013;15(12):941–7. 10.1038/gim.2013.41 23579436

[11] Peixoto A, Pinheiro M, Massena L, Santos C, Pinto P, Rocha P, et al Genomic characterization of two large Alu-mediated rearrangements of the BRCA1 gene. J Hum Genet. 2013;58(2):78–83. 10.1038/jhg.2012.137 23223007

[12] Rohlfs EM, Puget N, Graham ML, Weber BL, Garber JE, Skrzynia C, et al An Alu-mediated 7.1 kb deletion of BRCA1 exons 8 and 9 in breast and ovarian cancer families that results in alternative splicing of exon 10. Genes Chromosomes Cancer. 2000;28(3):300–7. 10862036 10.1002/1098-2264(200007)28:3<300::aid-gcc8>3.0.co;2-1

[13] Ross OA, Braithwaite AT, Skipper LM, Kachergus J, Hulihan MM, Middleton FA, et al Genomic investigation of alpha-synuclein multiplication and parkinsonism. Ann Neurol. 2008;63(6):743–50. Epub 2008/06/24. PubMed Central PMCID: PMC3850281. 10.1002/ana.21380 18571778 PMC3850281

[14] Bollati V, Schwartz J, Wright R, Litonjua A, Tarantini L, Suh H, et al Decline in genomic DNA methylation through aging in a cohort of elderly subjects. Mech Ageing Dev. 2009;130(4):234–9. PubMed Central PMCID: PMCPMC2956267. 10.1016/j.mad.2008.12.003 19150625 PMC2956267

[15] Jintaridth P, Tungtrongchitr R, Preutthipan S, Mutirangura A. Hypomethylation of Alu elements in post-menopausal women with osteoporosis. PLoS One. 2013;8(8):e70386 PubMed Central PMCID: PMCPMC3749148. 10.1371/journal.pone.0070386 23990903 PMC3749148

[16] Jintaridth P, Mutirangura A. Distinctive patterns of age-dependent hypomethylation in interspersed repetitive sequences. Physiol Genomics. 2010;41(2):194–200. 10.1152/physiolgenomics.00146.2009 20145203

[17] Wildschutte JH, Baron A, Diroff NM, Kidd JM. Discovery and characterization of Alu repeat sequences via precise local read assembly. Nucleic Acids Res. 2015;43(21):10292–307. PubMed Central PMCID: PMCPMC4666360. 10.1093/nar/gkv1089 26503250 PMC4666360

[18] Oler AJ, Traina-Dorge S, Derbes RS, Canella D, Cairns BR, Roy-Engel AM. Alu expression in human cell lines and their retrotranspositional potential. Mob DNA. 2012;3(1):11 PubMed Central PMCID: PMCPMC3412727. 10.1186/1759-8753-3-11 22716230 PMC3412727

[19] Hormozdiari F, Alkan C, Ventura M, Hajirasouliha I, Malig M, Hach F, et al Alu repeat discovery and characterization within human genomes. Genome Res. 2011;21(6):840–9. PubMed Central PMCID: PMCPMC3106317. 10.1101/gr.115956.110 21131385 PMC3106317

[20] Kim YH, Hong SJ, Jung YC, Kim SJ, Seo EJ, Choi SW, et al The 5'-end transitional CpGs between the CpG islands and retroelements are hypomethylated in association with loss of heterozygosity in gastric cancers. BMC Cancer. 2006;6:180 PubMed Central PMCID: PMCPMC1552088. 10.1186/1471-2407-6-180 16827945 PMC1552088

[21] Daskalos A, Nikolaidis G, Xinarianos G, Savvari P, Cassidy A, Zakopoulou R, et al Hypomethylation of retrotransposable elements correlates with genomic instability in non-small cell lung cancer. Int J Cancer. 2009;124(1):81–7. 10.1002/ijc.23849 18823011

[22] Chen J, Gong M, Lu S, Liu F, Xia L, Nie D, et al Detection of serum Alu element hypomethylation for the diagnosis and prognosis of glioma. J Mol Neurosci. 2013;50(2):368–75. 10.1007/s12031-013-0014-8 23657981

[23] Burnet FM. The immunological significance of the thymus: an extension of the clonal selection theory of immunity. Australas Ann Med. 1962;11:79–91. 13874950 10.1111/imj.1962.11.2.79

[24] Nowell PC. The clonal evolution of tumor cell populations. Science. 1976;194(4260):23–8. 959840 10.1126/science.959840

[25] Mustafina OE. The possible roles of human Alu elements in aging. Front Genet. 2013;4:96 PubMed Central PMCID: PMCPMC3664780. 10.3389/fgene.2013.00096 23755069 PMC3664780

[26] Venturelli M, Morgan GR, Donato AJ, Reese V, Bottura R, Tarperi C, et al Cellular aging of skeletal muscle: telomeric and free radical evidence that physical inactivity is responsible and not age. Clin Sci (Lond). 2014;127(6):415–21. 24708050 10.1042/CS20140051 PMC4757470

[27] Karlen Y, McNair A, Perseguers S, Mazza C, Mermod N. Statistical significance of quantitative PCR. BMC Bioinformatics. 2007;8:131 PubMed Central PMCID: PMCPMC1868764. 10.1186/1471-2105-8-131 17445280 PMC1868764

[28] Cawthon RM. Telomere length measurement by a novel monochrome multiplex quantitative PCR method. Nucleic Acids Res. 2009;37(3):e21 PubMed Central PMCID: PMCPMC2647324. 10.1093/nar/gkn1027 19129229 PMC2647324

[29] Morgan RG, Ives SJ, Lesniewski LA, Cawthon RM, Andtbacka RH, Noyes RD, et al Age-related telomere uncapping is associated with cellular senescence and inflammation independent of telomere shortening in human arteries. Am J Physiol Heart Circ Physiol. 2013;305(2):H251–8. Epub 2013/05/15. PubMed Central PMCID: PMC3726958. 10.1152/ajpheart.00197.2013 23666675 PMC3726958

[30] Morgan RG, Ives SJ, Walker AE, Cawthon RM, Andtbacka RH, Noyes D, et al Role of arterial telomere dysfunction in hypertension: relative contributions of telomere shortening and telomere uncapping. J Hypertens. 2014;32(6):1293–9. Epub 2014/04/02. PubMed Central PMCID: PMC4198301. 10.1097/HJH.0000000000000157 24686009 PMC4198301

[31] Colegrave N, Ruxton GD. Confidence intervals are a more useful complement to nonsignificant tests than are power calculations. Behavioral Ecology. 2003;14(3):446–7.

[32] Cumming G, Finch S. Inference by eye: confidence intervals and how to read pictures of data. The American psychologist. 2005;60(2):170–80. Epub 2005/03/03. 10.1037/0003-066X.60.2.170 15740449

[33] Knol MJ, Pestman WR, Grobbee DE. The (mis)use of overlap of confidence intervals to assess effect modification. Eur J Epidemiol. 2011;26(4):253–4. Epub 2011/03/23. PubMed Central PMCID: PMCPMC3088813. 10.1007/s10654-011-9563-8 21424218 PMC3088813

[34] Cumming G, Finch S. A Primer on the Understanding, Use, and Calculation of Confidence Intervals that are Based on Central and Noncentral Distributions. Educational and Psychological Measurement. 2001;61(4):532–74.

[35] Steenstrup T, Hjelmborg JV, Mortensen LH, Kimura M, Christensen K, Aviv A. Leukocyte telomere dynamics in the elderly. Eur J Epidemiol. 2013;28(2):181–7. PubMed Central PMCID: PMCPMC3604590. 10.1007/s10654-013-9780-4 23430034 PMC3604590

[36] Kimura M, Barbieri M, Gardner JP, Skurnick J, Cao X, van Riel N, et al Leukocytes of exceptionally old persons display ultra-short telomeres. Am J Physiol Regul Integr Comp Physiol. 2007;293(6):R2210–7. 10.1152/ajpregu.00615.2007 17898116

[37] Daniali L, Benetos A, Susser E, Kark JD, Labat C, Kimura M, et al Telomeres shorten at equivalent rates in somatic tissues of adults. Nat Commun. 2013;4:1597 PubMed Central PMCID: PMCPMC3615479. 10.1038/ncomms2602 23511462 PMC3615479

[38] Galkina E, Ley K. Immune and Inflammatory Mechanisms of Atherosclerosis*. Annual Review of Immunology. 2009;27(1):165–97. 10.1146/annurev.immunol.021908.132620 PMC2734407 19302038

